# Establishment and validation of a nomogram model for aseptic loosening after tumor prosthetic replacement around the knee: a retrospective analysis

**DOI:** 10.1186/s13018-019-1423-3

**Published:** 2019-11-09

**Authors:** Hao-ran Zhang, Feng Wang, Xiong-gang Yang, Ming-you Xu, Rui-qi Qiao, Ji-kai Li, Yun-long Zhao, Cheng-gang Pang, Xiu-chun Yu, Yong-cheng Hu

**Affiliations:** 1 Department of Bone Tumor, Tianjin Hospital, 406 Jiefang Southern Road, Tianjin, China; 2 Graduate School, Tianjin Medical University, 22 Qixiangtai Road, Tianjin, China; 3 Department of Orthopedics, General Hospital of Jinan Military Commanding Region, 25 Shifan Road, Jinan, Shandong China

**Keywords:** Tumor prosthesis, Aseptic loosening, Risk factor, Nomogram model, Knee

## Abstract

**Background:**

Aseptic loosening has become the main cause of prosthetic failure in medium- to long-term follow-up. The objective of this study was to establish and validate a nomogram model for aseptic loosening after tumor prosthetic replacement around knee.

**Methods:**

We collected data on patients who underwent tumor prosthetic replacements. The following risk factors were analyzed: tumor site, stem length, resection length, prosthetic motion mode, sex, age, extra-cortical grafting, custom or modular, stem diameter, stem material, tumor type, activity intensity, and BMI. We used univariate and multivariate Cox regression for analysis. Finally, the significant risk factors were used to establish the nomogram model.

**Results:**

The stem length, resection length, tumor site, and prosthetic motion mode showed a tendency to be related to aseptic loosening, according to the univariate analysis. Multivariate analysis showed that the tumor site, stem length, and prosthetic motion mode were independent risk factors. The internal validation indicated that the nomogram model had acceptable predictive accuracy.

**Conclusions:**

A nomogram model was developed for predicting the prosthetic survival rate without aseptic loosening. Patients with distal femoral tumors and those who are applied with fixed hinge and short-stem prostheses are more likely to be exposed to aseptic loosening.

## Introduction

The knee joint is a common site for primary and metastatic bone tumors, with reported incidences of 28.4% for benign bone tumors and 46.84% for malignant bone tumors [1]. In the recent decades, the treatment for malignant bone tumors around knee has shifted from amputation to limb salvage surgery, in which the tumor prostheses are most commonly selected for the reconstruction of bone defects [2–4]. Although many advantages could be provided by tumor prostheses over other reconstructive methods, including the easier availability, earlier weight bearing, and acceptable appearance, there are still some patients who require revision surgery due to various complications. Among them, aseptic loosening is one of the most frequently encountered long-term complications after prosthetic replacement, giving decreased service life of prosthesis and increased economic and adverse emotional burdens to patients substantially.

A multicentric retrospective study for 2174 mature patients treated with artificial prosthetic replacements following tumor resection has shown that aseptic loosening occurred later (76 months) than other types of failure modes with the highest incidence among mechanical failures [5]. Unwin et al. [6] reported that the aseptic loosening free survival rates of tumor prosthesis were only 67% in the group with distal femoral tumors and 55% in the group with proximal tibial tumors at a 10-year follow-up, respectively, indicating that the aseptic loosening bears crucial responsibility for the long-term failure of tumor prosthesis. However, few authors have specialized in the risk factors for aseptic loosening and an easy-to-use prediction model has not been built up.

The nomogram model, which transforms traditional statistical predictive models into visualized probability estimates tailored to each patient, is suitable for cancer prognostic studies. This kind of user-friendly graphical representation can allow for clinicians to more easily explain prognosis information to patients, rather than abstractly presenting them with risk factors. In addition, the individualized prediction of the nomogram makes it possible to identify and stratify patients involved in clinical trials [7].

The purposes of this study were to identify the risk factors of aseptic loosening for patients treated with tumor prosthesis and establish and validate a nomogram model which can assist clinicians and patients in predicting the aseptic loosening free survival following reconstruction with tumor prosthesis.

## Patients and methods

### Inclusion and exclusion criteria

This research was conducted according to the “Transparent Reporting of a multivariable prediction model for Individual Prognosis or Diagnosis (TRIPOD)” statement [8]. Data on patients who underwent tumor prosthetic replacements between August 2001 and September 2016 were retrospectively collected from two clinical centers. Tumor prosthetic replacements were carried out for management of reconstruction following resection of malignant bone tumors and invasive (stage 3) benign bone tumors. The inclusion criteria were the use of tumor prostheses for primary reconstruction of the mega bone defect after tumor resection. The exclusion criteria were extendable prostheses used for children, uncemented prostheses, allograft-prosthetic composites, and patients who were lost to follow up. This study followed the “Declaration of Helsinki” and was approved by the hospital ethics committee and obtained informed consent from the patients.

### Surgical procedures and prosthesis

The medial or lateral arc incision was selected to expose the knee joint and made an elliptical incision around the biopsy scar to remove the biopsy channel. The medial and lateral collateral ligaments, cruciate ligaments, and meniscus were severed to dislocate the knee joint. The osteotomy plane was determined by where the normal bone marrow signal on T1WI becomes an abnormal signal. Resection of tumor was based on common oncology principles [9]. The marrow cavity of the distal femur and proximal tibia was expanded until it was 2 mm larger than the diameter of prosthetic stem. The trial prosthesis was then placed to adjust the condition of surrounding soft tissue and force line and for activity tracking of the patella. The medullary cavity was washed and injected with bone cement, the proximal and distal prosthetic stems were placed, and additional components were installed. We usually did not perform patella replacement. Reconstruction of knee extension mechanism involved suturing the patellar tendon with non-absorbable suture on the anterior hole of the prosthesis or the gastrocnemius. If the soft tissue envelope was difficult to suture, free skin grafting was considered to reduce tension. We used two kinds of native tumor prostheses (Lidakang, Beijing, China and Wego, Beijing, China). Neither of them possessed a hydroxyapatite (HA) collar design.

### Recorded data

Demographic, preoperative, intraoperative, and postoperative data were obtained from medical records and subsequent follow-up. Preoperative data included the tumor site (distal femur vs. proximal tibia), sex (female vs. male), age (> 30 years vs. ≤ 30 years), prosthetic motion mode (fixed vs. rotating hinge), material for prosthetic stem (CoCrMo vs. titanium), type of prosthesis (custom vs. modular), and type of tumor (benign vs. malignant). Intraoperative data included length of prosthetic stem (≥ 14 cm vs. < 14 cm), length of bone resection (≥ 14 cm vs. < 14 cm), extra-cortical grafting (yes vs. no), and diameter of prosthetic stem (≥ 13 mm vs. < 13 mm). Postoperative data included the intensity of activity (high vs. low) and body mass index (BMI) (≥ 25 vs. < 25). These previously described factors were included as explanatory variables in this study. The calculation of resection length at the distal femur and proximal tibia was based on the femoral condyle and tibial plateau, respectively. Patients who were retired at home or were involved in limited leisure activities were described as low-intensity, and those who regularly participated in physical exercise were categorized as high-intensity. In terms of diameter of prosthetic stem, length of prosthetic stem, and length of bone resection, patients were divided into two groups about the mean value for each independent factor. The response variable was aseptic loosening, which was defined as fracture of the cement mantle or progressive radiolucencies between bone-prosthesis interface, without infection.

### Statistical analysis

Descriptive statistics were calculated using means for continuous data and proportions for count data. Survival analysis was performed with Kaplan-Meier curves [10], and patients were censored for any other cause of failure, death, and no problem at latest follow-up. To determine the risk factors associated with aseptic loosening, univariate Cox regression analysis was carried out, and variables significant at the *p* ≤ 0.15 level were included in the multivariate Cox regression analysis [11]. *p* values were based on two-tailed tests and at < 0.05 were considered significant. The forest plot was used to display the results of univariate and multivariate analyses. Finally, a nomogram model was constructed using the independent risk factors of the multivariate analysis. The concordance index (C-index), which ranged from 0.5 to 1.0, was applied to validate the discrimination, and then the calibration curve was applied to validate the consistence for each time point. The building, validation, and interpretations of the nomogram model were carried out following the literature published by Iasonos et al. [7]. Statistical analysis was performed using R version 3.5.1 for Windows (R Foundation for Statistical Computing, Vienna, Austria), SPSS 22.0 software (SPSS Inc., Chicago, Illinois, USA), and GraphPad Prism 7 Software (GraphPad Software Inc., San Diego, CA).

## Results

### Baseline characteristics

Two hundred and twenty-eight prostheses were identified. Eighteen implants were excluded because they were allograft-prosthetic composites or extendable prostheses and 23 prostheses had been lost to follow-up, leaving 177 prostheses for evaluation. There were 61 female patients (34.5%) and 116 male patients (65.5%). The mean age at the time of surgery was 35 years (range, 13–77 years). One hundred nineteen and 58 implants were placed in the distal femur and proximal tibia, respectively. The histologic diagnoses were osteosarcoma in 85 patients, giant cell tumor in 61 patients, malignant fibrous histiocytoma in 14 patients, chondrosarcoma in 9 patients, and other sarcomas in 8 patients. Table 1 displays the baseline information including type of prosthesis, tumor site, and additional details of the patients and prostheses.

**Table 1:** Baseline characteristics of the patients and prostheses

Variables	Number	Percent of relevant group
Sex		
Female	61	34.5
Male	116	65.5
Age		
> 30 years	95	53.7
≤ 30 years	82	46.3
Type of prosthesis		
Custom	103	58.2
Modular	74	41.8
Tumor site		
Distal femur	119	67.2
Proximal tibia	58	32.8
Prosthetic motion mode		
Fixed hinge	19	10.7
Rotating hinge	158	89.3
Type of tumor		
Osteosarcoma	85	48.0
Giant cell tumor	61	34.5
Malignant fibrous histiocytoma	14	7.9
Chondrosarcoma	9	5.1
Other sarcomas	8	4.5
Primary malignant tumor staging		
IIA	23	19.8
IIB	57	49.1
III	36	31.0

### Clinical status

Minimum duration of follow-up was 2 years (mean, 92 months; range, 24–199 months). At the most recent follow-up, among the 177 patients with benign or malignant bone tumors, 121 were continuously disease-free, 23 had no disease after treatment of local recurrence, nine were alive with tumor, and 24 were deceased. Of 116 patients with sarcomas, 11 (9.5%) developed a recurrence, and of those, 27 (23.3%) were thought to exhibit metastasis. All patients with giant cell tumor were alive.

The incidence of prosthetic failure in our study was 24.9% (44 of 177). Mechanical failures occurred in 61.4% (27 of 44) of all failures. Only two (4.5%) of all failures were due to problems related to soft tissues, and most treatment measures did not involve the prosthesis itself. Twenty-two failures (50%) were from aseptic loosening, and three failures (6.8%) were due to periprosthetic or prosthetic fractures. Nonmechanical failures accounted for 38.6% (17 of 44) of all failures, which included five failures (11.4%) that were due to tumor progression and 12 failures (34%) that came from deep infection. The absolute risk of every failure mode type 1 through 5 was 1.1%, 12.4%, 1.7%, 6.8%, and 2.8%, respectively.

### Aseptic loosening

**Fig. 1:**
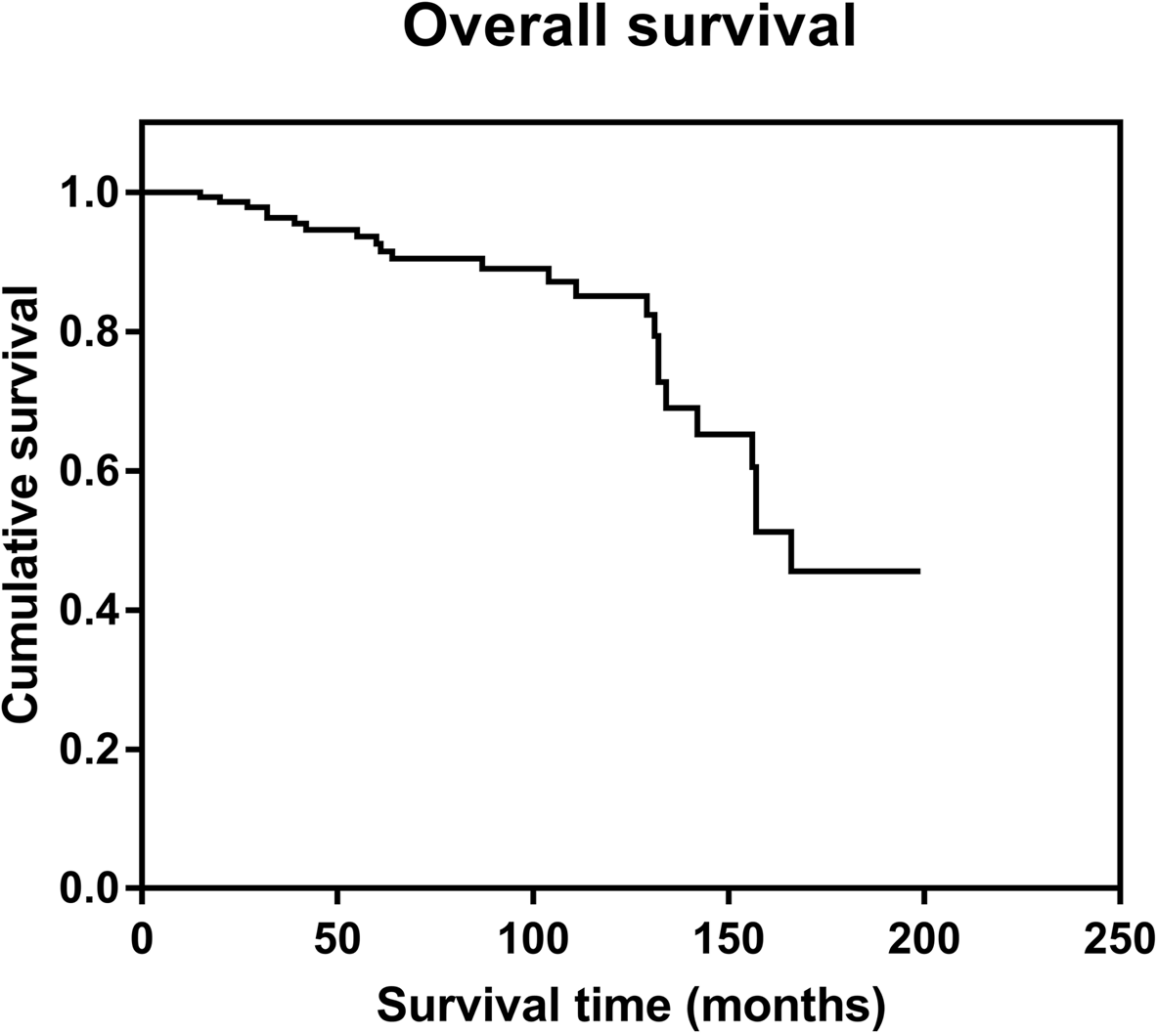
The Kaplan-Meier survival curve for the whole cohort. The prosthetic survival rate without aseptic loosening is 92.5% at 5 years, 85.0% at 10 years, and 45.5% at 15 years

Aseptic loosening occurred in 13.6% (24 of 177) prostheses at mean of 91 months (range, 15–166 months). The prosthetic survival rate without aseptic loosening was 92.5% at 5 years, 85.0% at 10 years, and 45.5% at 15 years (Fig. 1). The incidence of aseptic loosening was 16.0% (19 of 119) in the distal femoral prostheses and 8.6% (5 of 58) in the proximal tibial prostheses. Rotating hinge prostheses (8.2%, 13 of 158) had a lower aseptic loosening rate than fixed hinge prostheses (57.9%, 11 of 19).

Most patients (22 of 24) with aseptic loosening achieved satisfactory results through revision surgery, and the remaining two patients were waiting for revision. During revision surgery, we reset the original prosthesis or replaced it with another prosthesis. The new prosthesis generally had a longer and thicker prosthetic stem than the original prosthesis.

### Risk factors associated with aseptic loosening

**Fig. 2:**
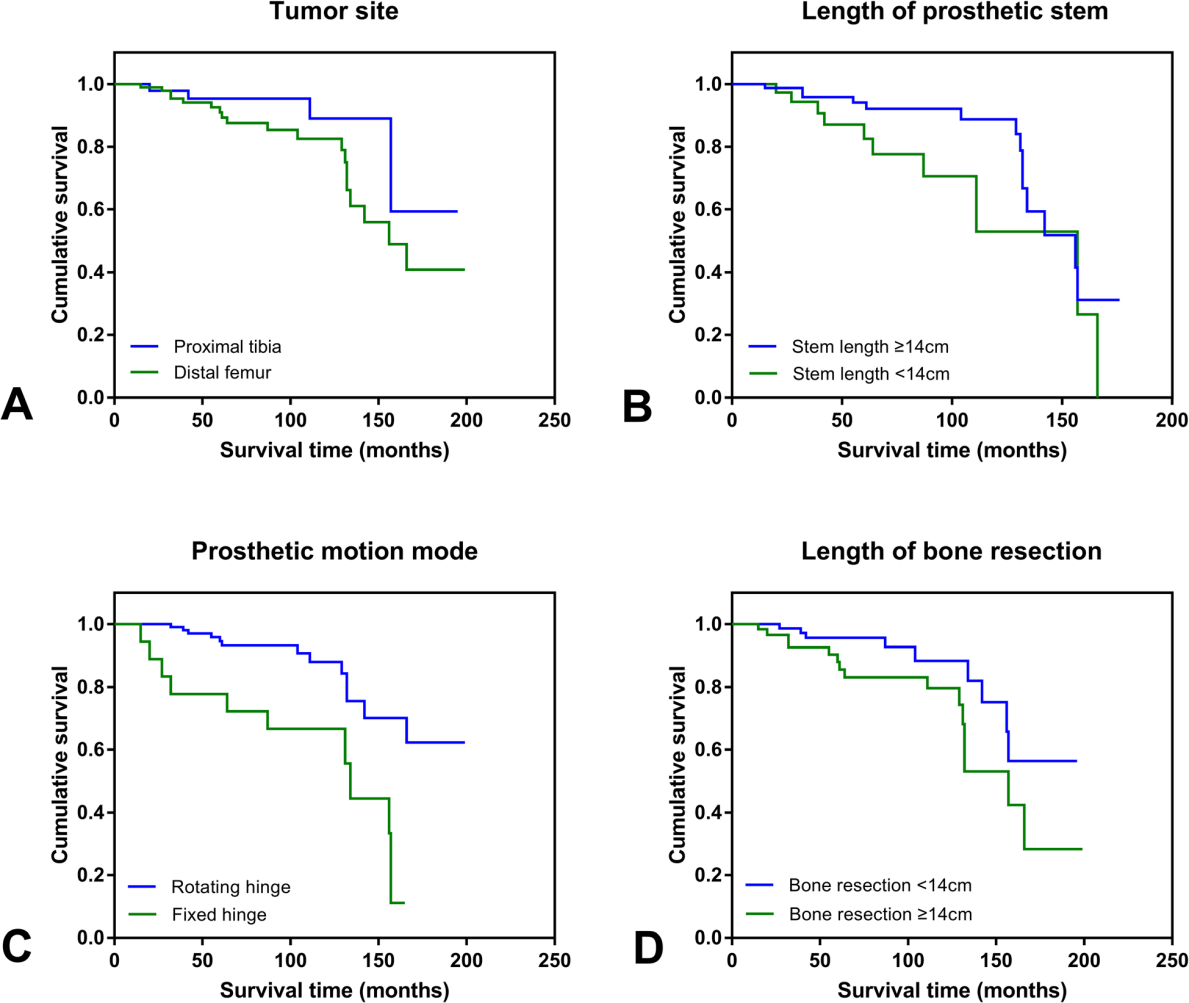
**a**–**d** The Kaplan-Meier survival curves for factors identified to be associated with aseptic loosening, including the tumor site (**a**), length of prosthetic stem (**b**), prosthetic motion mode (**c**), and length of bone resection (**d**)

**Fig. 3:**
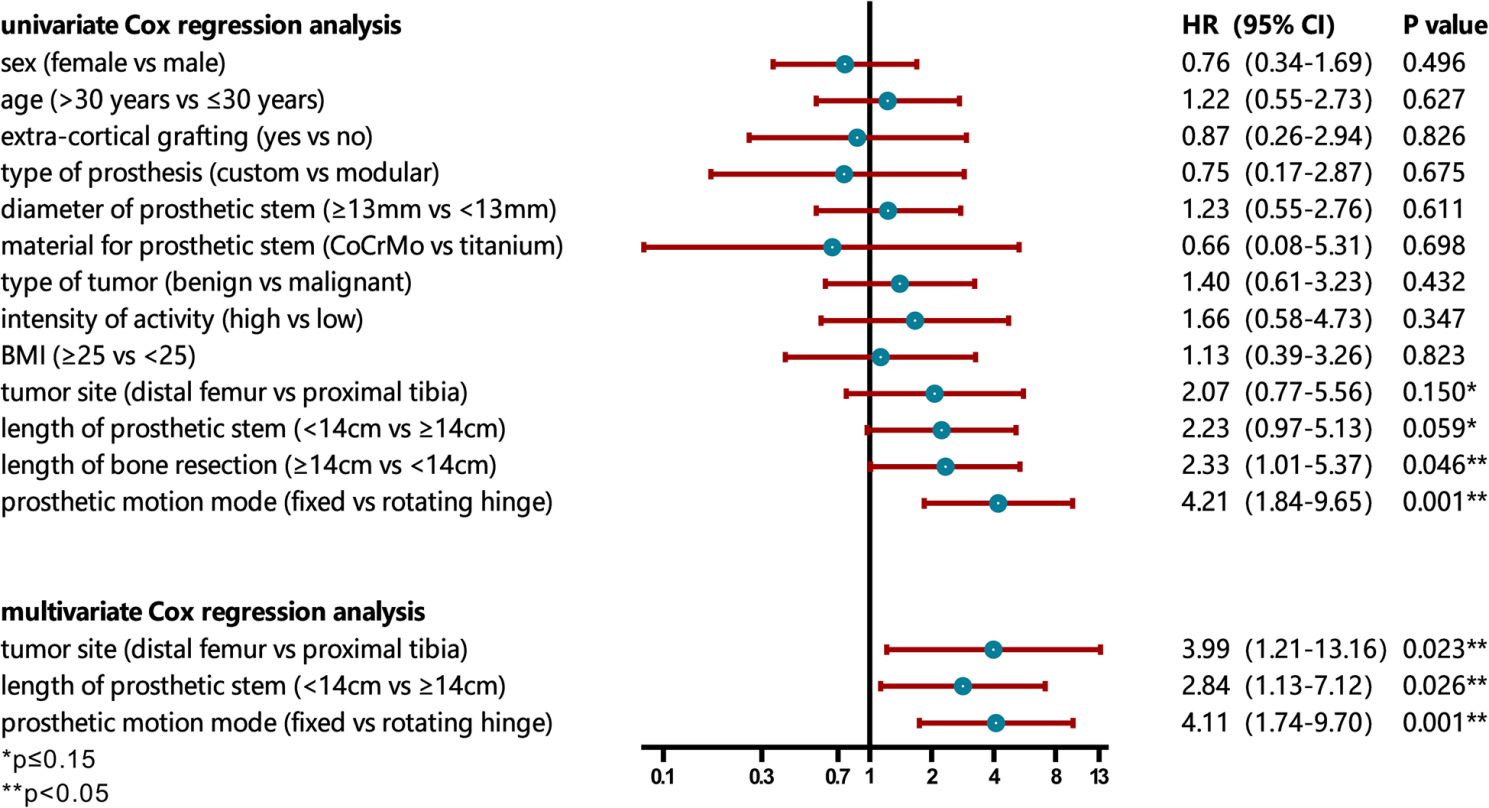
The forest plot shows the results of univariate and multivariate analyses. The length of bone resection (*p* = 0.046), length of prosthetic stem (*p* = 0.059), prosthetic motion mode (*p* = 0.001), and tumor site (*p* = 0.150) are demonstrated to be related to aseptic loosening in univariate analysis. While in multivariable analysis, the tumor site (HR = 3.99, CI 95% 1.21~13.16, *p* = 0.023), length of prosthetic stem (HR = 2.84, CI 95% 1.13~7.12, *p* = 0.026), and prosthetic motion mode (HR = 4.11, CI 95% 1.74~9.70, *p* = 0.001) are independent risk factors for aseptic loosening

Univariate Cox regression analysis showed that the length of bone resection (*p* = 0.046) and prosthetic motion mode (*p* = 0.001) were risk factors for aseptic loosening. The length of prosthetic stem (*p* = 0.059) and tumor site (*p* = 0.150) were shown to be marginally significant for predicting aseptic loosening (Fig. 2). Other factors including the sex (*p* = 0.496), type of tumor (*p* = 0.432), age (*p* = 0.627), extra-cortical grafting (*p* = 0.826), custom or modular (*p* = 0.675), diameter of prosthetic stem (*p* = 0.611), material for prosthetic stem (*p* = 0.698), intensity of activity (*p* = 0.347), and BMI (*p* = 0.823) were not risk factors for aseptic loosening of tumor prosthesis (Table 2; Fig. 3).

**Table 2:** Univariate and multivariate analyses of risk factors for aseptic loosening

Variables	Number of cases	Survival rate (%)		Mean survival time (months)	*p* value	Multivariate analysis		
		5 years	10 years			HR	95% CI	*p* value
Sex								
Female	61	89.6	85.8	163.1				
Male	116	96.9	84.6	149.9	0.496			NI
Age								
> 30 years	95	92.8	83.6	153.4				
≤ 30 years	82	92.6	86.4	161.0	0.627			NI
Extra-cortical grafting								
Yes	17	86.2	78.3	167.0				
No	160	93.5	85.8	155.2	0.826			NI
Type of prosthesis								
Custom	103	93.4	85.5	158.8				
Modular	74	88.4	NA	84.9	0.675			NI
Diameter of prosthetic stem								
≥ 13 mm	72	88.8	74.5	137.7				
< 13 mm	74	92.6	88.9	154.0	0.611			NI
Material for prosthetic stem								
CoCrMo	56	92.9	NA	77.3				
Titanium	117	92.0	84.3	157.0	0.698			NI
Type of tumor								
Benign	61	100	84.6	152.5				
Malignant	116	88.4	86.6	171.5	0.432			NI
Intensity of activity								
High	46	86.7	NA	96.3				
Low	94	90.3	63.7	108.9	0.347			NI
BMI								
≥ 25	105	89.9	72.5	116.5				
< 25	50	93.0	88.6	121.3	0.823			NI
Tumor site								
Distal femur	119	90.9	82.5	151.8		3.99		
Proximal tibia	58	95.4	89.0	170.8	0.150	1	(1.21, 13.16)	0.023
Length of prosthetic stem								
≥ 14 cm	73	94.1	88.8	143.5		1		
< 14 cm	75	82.5	52.9	121.4	0.059	2.84	(1.13, 7.12)	0.026
Length of bone resection								
≥ 14 cm	82	88.0	79.6	142.7				
< 14 cm	89	95.6	88.3	166.8	0.046			0.382
Prosthetic motion mode								
Fixed hinge	19	77.8	66.7	113.6		4.11		
Rotating hinge	158	94.6	88.0	170.0	0.001	1	(1.74, 9.70)	0.001

*NI* not included, *NA* not available

Multivariate Cox regression analysis indicated that the tumor site (HR = 3.99, CI 95% 1.21~13.16, *p* = 0.023), length of prosthetic stem (HR = 2.84, CI 95% 1.13~7.12, *p* = 0.026), and prosthetic motion mode (HR = 4.11, CI 95% 1.74~9.70, *p* = 0.001) were independent risk factors for aseptic loosening (Table 2; Fig. 3). The length of bone resection (*p* = 0.382) was excluded from the model.

### Nomogram model

**Fig. 4:**
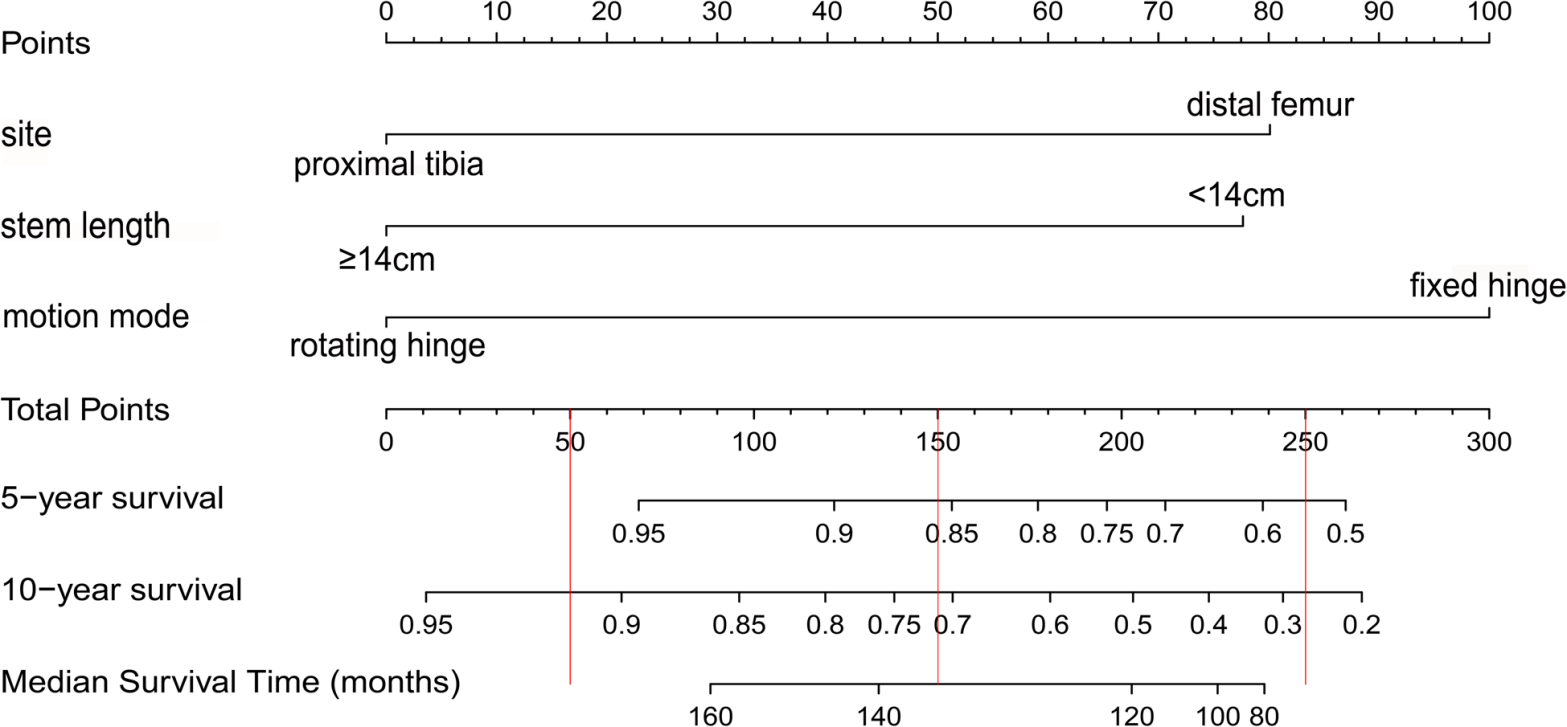
A nomogram predictive model for aseptic loosening after tumor prosthetic replacement around the knee joint. The corresponding points for each factor are based on the condition of the patient, which can be determined by making a vertical line upwards (e.g., a patient with prosthetic stem length < 14 cm will receive between 70 and 80 points). Add all the points to get the total score, then find the corresponding points on the total points axis and make a vertical line down to predict the 5-year and 10-year survival rate without aseptic loosening

**Fig. 5:**
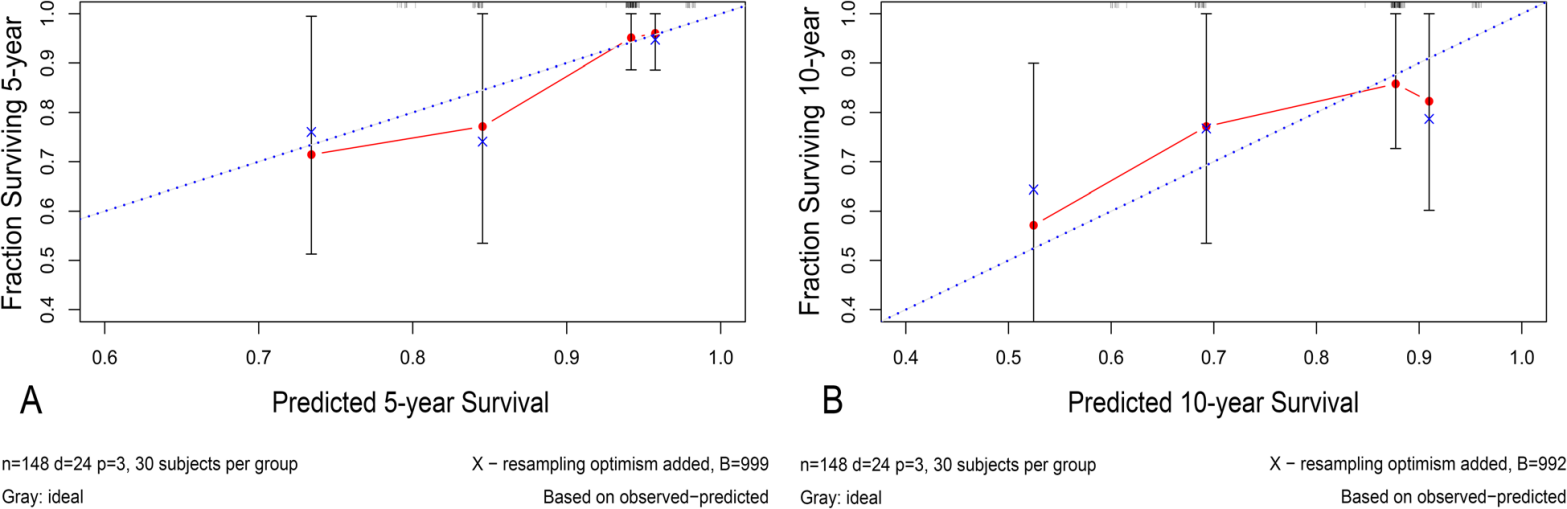
**a**–**b** The calibration curves for assessing the consistency between the predicted and the actual prosthetic survival rate without aseptic loosening. A good consistency between the predicted and the actual survival probabilities is presented, for time points of 5 years (**a**) and 10 years (**b**)

A nomogram model was constructed based on the three independent risk factors derived from the multivariate analysis (Fig. 4). Each factor in the model was given a weighted point. The sum of each factor was the total points of the patient. According to the total points, the prosthetic survival rate without aseptic loosening for 5 years, 10 years, and median survival time can be calculated. The greater the number of points, the lower the prosthetic survival rate without aseptic loosening. The C-index was 0.74 (CI 95% 0.65~0.84), which indicated that the nomogram model had acceptable predictive discrimination. The calibration curves for time points of 5 years and 10 years are shown in Fig. 5. There was a good consistency between the predicted and the actual prosthetic survival rates shown for each time point.

## Discussion

Endoprosthetic replacements prevent bone tumor patients from needing amputation, but there are risks of failure and revision. Aseptic loosening is the most common cause of prosthetic failure during long term follow-up [12, 13], and it increases with the prolonging of implanting time whether the prosthetic stem is cemented or uncemented [5, 6, 12, 14–16]. Due to differences in tumor site, fixation technique, and type of prosthesis, the incidence of aseptic loosening varies widely among studies, ranging from 0 to 27.5% [4, 5, 12, 14, 17–29]. The current study showed that the tumor site, stem length, and prosthetic motion mode were independent risk factors for aseptic loosening. A nomogram model was established using these significant predictors, with a C-index of 0.74 and a favorable consistency between predicted and actual aseptic loosening rate according to the internal validation, indicating that the nomogram model had acceptable predictive accuracy.

Some unchangeable factors, such as tumor site, may affect the aseptic loosening rate of tumor prosthesis. The incidence of aseptic loosening is 5.3–14.6% in the distal femur and 0–7.1% in the proximal tibia [5, 21, 27, 30]. Pala et al. [31] studied 295 lower limb prostheses, including 199 cases of distal femur, 60 cases of proximal tibia, 32 cases of proximal femur, and four cases of total femur. They found that the distal femoral prostheses had the highest aseptic loosening rate in all areas. Similarly, Mittermayer et al. [32] reported the distal femur had the highest aseptic loosening rate, followed by the proximal tibia and proximal femur (*p* = 0.05). Our study supports the view of most literature that distal femoral prosthesis has a higher aseptic loosening rate (HR = 3.99, CI 95% 1.21~13.16, *p* = 0.023). Due to differences in anatomical structure, the distal femoral prosthesis has a larger offset from the force line than proximal tibia, resulting in a larger torque. And the cross section of the medullary cavity of the tibia is close to the triangle rather than the round shape of the femur, so the prosthetic stem of proximal tibial prosthesis is easier to stabilize [2].

It is generally accepted that a rotating hinge structure helps reduce the incidence of aseptic loosening [2, 12]. The introduction of the rotating hinge structure allows flexion-extension, external-internal rotation, and proximal-distal translation (distraction), therefore decreasing bushing wear and transferring the stress from the stem to the joint [21, 24, 28, 33, 34]. In contrast, stress cannot appropriately distribute around the knee in the patients who use fixed hinge prostheses, and this may cause subsequent aseptic loosening [12, 26]. Myers et al. [16] described 194 cases of proximal tibial prostheses and found that the aseptic loosening rate at 10 years of fixed hinge prostheses (46%) was higher than that of rotating hinge prostheses (3%) (*p* < 0.0001). Our study supports this conclusion, and multivariate analysis reveals that fixed hinge prosthesis has more than a fourfold risk of developing aseptic loosening than rotating hinge prosthesis (HR = 4.11, CI 95% 1.74~9.70, *p* = 0.001).

The degree of medullary fit and fill, which involves length, curvature, and diameter of the intramedullary stem, is an important factor affecting the stability of the prosthesis [35]. Batta et al. [17] noted that the increased TPL/SL (total length of prosthesis/stem length) ratio will increase the risk of aseptic loosening. Bergin et al. [18] studied 104 cemented modular prostheses with an average of 5.6 years follow-up to explore the factors affecting aseptic loosening and noted that the greater bone/stem ratio predicted the higher risk of aseptic loosening. We can determine that the length of intramedullary stem is an independent risk factor for aseptic loosening (HR = 2.84, CI 95% 1.13~7.12, *p* = 0.026), but the diameter is not (*p* = 0.611). This suggests that, under the background of modular prostheses are now widely applied, surgeons should try to choose a longer prosthetic stem to fully fill the medullary cavity.

We believe that the nomogram model has great clinical value for individualized medicine. Through the nomogram model, clinicians can introduce complex statistical analysis results to patients and improve patient compliance. Clinicians should make cooperative efforts with patients to avoid aseptic loosening and improve quality of life. This model requires additional prospective research and external validation to further confirm its accuracy.

We acknowledge that the current study has limitations. This is a non-random retrospective study which may produce choice and recall bias. Moreover, due to the small number of uncemented prostheses in our institutions, we were unable to accurately analyze the difference in aseptic loosening between the two kinds of fixation method. And we consider that having a porous or HA coated collar is a potentially important factor affecting survival of tumor implants, but the native prosthesis we used did not have this specific design. Lastly, external validation should be conducted in a cohort of patients from different regions and ethnicities to verify the broad applicability of this model.

## Conclusion

In conclusion, our findings suggest that doctors should inform patients with distal femoral tumors of the high risk of aseptic loosening in the future and choose a rotating hinge, long-stem prosthesis during endoprosthetic replacement if the conditions allow. A nomogram model, which creates a concise graphical representation that produces a numerical prosthetic survival rate, was established using the significant risk factors. Internal validation was performed, which indicated an acceptable discrimination and consistency. Doctors can apply the user-friendly model tailored to the profile of each individual patient to explain risk factors and related precautions to patients.

## Data Availability

The datasets used and/or analyzed during the current study are available from the corresponding author on reasonable request.

## Abbreviations

BMI: Body mass index
C-index: Concordance index
HA: Hydroxyapatite
